# Design and Characterization of a Fully Differential MEMS Accelerometer Fabricated Using MetalMUMPs Technology

**DOI:** 10.3390/s130505720

**Published:** 2013-05-02

**Authors:** Peng Qu, Hongwei Qu

**Affiliations:** Department of Electrical and Computer Engineering, Oakland University, SEB 102n 2200 N. Squirrel Road, Rochester, MI 48309, USA; E-Mail: pqu@oakland.edu

**Keywords:** accelerometer, MetalMUMPs, capacitive sensor, fully differential sensing

## Abstract

This paper presents a fully differential single-axis accelerometer fabricated using the MetalMUMPs process. The unique structural configuration and common-centriod wiring of the metal electrodes enables a fully differential sensing scheme with robust metal sensing structures. CoventorWare is used in structural and electrical design and simulation of the fully differential accelerometer. The MUMPs foundry fabrication process of the sensor allows for high yield, good process consistency and provides 20 μm structural thickness of the sensing element, which makes the capacitive sensing eligible. In device characterization, surface profile of the fabricated device is measured using a Veeco surface profilometer; and mean and gradient residual stress in the nickel structure are calculated as approximately 94.7 MPa and −5.27 MPa/μm, respectively. Dynamic characterization of the sensor is performed using a vibration shaker with a high-end commercial calibrating accelerometer as reference. The sensitivity of the sensor is measured as 0.52 mV/g prior to off-chip amplification. Temperature dependence of the sensing capacitance is also characterized. A −0.021fF/°C is observed. The findings in the presented work will provide useful information for design of sensors and actuators such as accelerometers, gyroscopes and electrothermal actuators that are to be fabricated using MetalMUMPs technology.

## 1. Introduction

One of the current trends in physical sensor technologies is hybrid rather than monolithic integration for optimal sensing element and dedicated electronics, and overall low cost and better performance [1]. Electroplated metal as sensing structural material has been attempted for robust sensors [2–5]. For process controllability and overall low cost of MEMS elements, foundry services are also preferable. Among the many MEMS foundry services, MUMPs provides relatively mature technologies for a variety of MEMS materials including metal. Various types of actuators fabricated using MetalMUMPs technology have been reported, of which most take great advantage of the thick nickel structural layer for large actuation force and displacement [6]. Recently metal sensors such as capacitive gyroscopes have been explored using MetalMUMPs or other adapted processes [2,3,7–9]. Compared to polysilicon sensing structures, the metal sensing elements, which normally have thicker structures, allow for larger proof mass, greater sensing capacitance, therefore smaller overall device size with comparable performance. In the reported metal gyroscope [2,3,7], the larger proof mass results in higher Coriolis force and increases the sensitivity in microgyroscope design. Although MetalMUMPs technology is widely used in actuators, due to constrains of its design rules, only few capacitive sensors have been attempted using this process [4,5,10]. In these reported displacement sensing scheme, a half-bridge capacitive sensing circuit is constructed using sidewall capacitance formed by nickel electrodes.

In this paper, a MetalMUMPs capacitive accelerometer employing in-plane sensing mechanism has been designed, fabricated and characterized. The motivation is to exploit some particular merits in MetalMUMPs technology, such as the excellent elastic and electrical properties of nickel, good process controllability, robust metal structure, low wiring parasitics and low cost potential. The uniqueness of this demonstrated device is the implementation of a fully differential sensing scheme with common-centroid wiring of the symmetrically partitioned sensing capacitor groups which also allows offset cancelation for large sensitivity. In device design, special connections are employed by using the doped polysilicon and insulator layers in the stacked structure, to realize mechanical connection and electrical isolation of the separated proof mass. To validate the unique sensing mechanism, a commercially available universal capacitive readout IC MS3110 from Irvine Sensor (Costa Mesa, CA, USA) was used in device characterizations.

Device characterization included derivation of residual stress in the structural nickel layer based on optical measurement of the fabricated devices, and electrical tests of the packaged device. A Veeco surface profilometer (Tucson, AZ, USA) was used in the optical measurement. Due to the robustness of the metal structures and particular design rules, MetalMUMPs sensors and actuators normally have larger dimensions than their polysilicon or single-crystal silicon counterparts. The undesired accumulated deformations of the structures could be significant in many MetalMUMPs devices. The residual stress characterized in this work can be a useful resource for MetalMUMPs users in design and optimization of their devices. The fully differential sensing scheme can also be applied to design of other capacitive devices.

## 2. Device Design and Simulation

**Figure 1.**
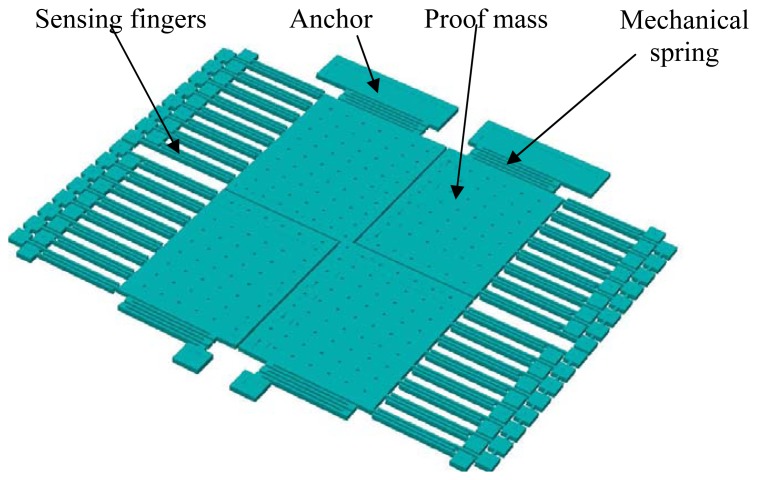
3-D structural model of the fully differential accelerometer.

Figure 1 shows a 3D model of the prototyped fully differential accelerometer fabricated using MetalMUMPs technology. The sensor has overall dimensions of approximately 1.6 mm × 1.2 mm in footprint, with a structural thickness of approximately 20 μm. The designed accelerometer consists of a 1.3 mm × 1.0 mm proof mass, sixty-four pairs of sensing comb drives, and four symmetrically folded mechanical springs through which the proof mass is anchored to the substrate. The equivalent dimension of each spring beam is 1,200 μm × 8 μm if stretched. In device design, MetalMUMPs design rules were strictly followed for successful structure release.

Table 1 summarizes critical technological parameters of MetalMUMPs technology that are related to device design and performance prediction [6]. The dimensions of the sensor structures and associated material properties are given in Table 2.

### 2.1. Device Structural Design

**Figure 2.**
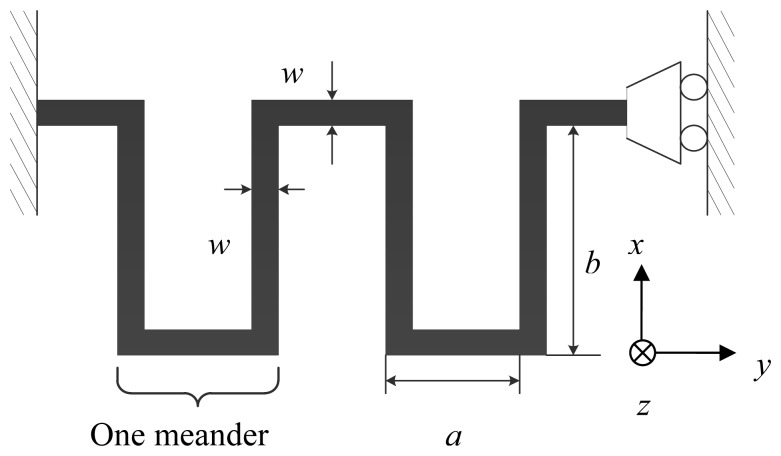
One of the four single folded springs used in the device.

As a typical second-order system, the mechanical performance of an accelerometer is largely determined by the response of its mechanical springs. To reduce the device size, folded mechanical springs have been used in the designed device, as shown in Figure 1. An accurate analytical model is used in mechanical spring design. The model has included effects of the stiffness of the meanders in the folded springs, as shown in Figure 2 [13].

Based on the configuration, the spring constant of the overall four guided-end springs can then be calculated as:

(1) $$k_{y}=\frac{48\text{EI}[(a+b)n-b]}{b^{2}(n-1)[(3a^{2}+4\text{ab}+b^{2})n+3a^{2}-b^{2}]}$$

where n is the number of meander, E is the Young's Modulus of nickel, the structural material in this device; a and b are geometrical parameters as shown in Figure 2. I = wt^3^/12 is moment of inertia of each spring beam which has a rectangular cross sections; w and t denotes the width and thickness of the springs, respectively.

The spring constant is then calculated as 73.6 N.m. With the given dimensions of the proof mass, the mechanical resonant frequency of the structure can be estimated as approximately 2.9 kHz.

### 2.2. Common-Centriod Wiring and Fully Differential Sensing

**Figure 3.**
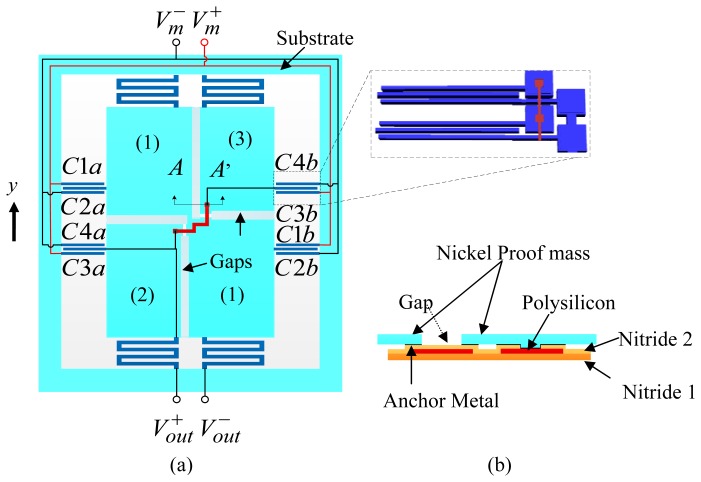
(**a**) Fully differential configuration of the accelerometer with inset showing the connection of sensing electrodes; and (**b**), cross-sectional view along AA' (in (a)) showing mechanical connections of the proof mass piece (1) and (3). The polysilicon underneath the gap is used for mechanical supporting.

As an exploratory study, this paper presents a fully differential capacitive sensing scheme implemented using MetalMUMPs process. A single-axis accelerometer with nickel structure is exemplified as a demonstrating device. Intuitively it is challenging to realize fully differential sensing schemes with metal microstructures because of the limits of the design rules for signal routing. All the reported MetalMUMPs capacitive sensors use a half-bridge sensing scheme [4,5,10]. The unique features of the accelerometer demonstrated in this work include a common-centriod wiring configuration for fully differential sensing scheme; and metal proof mass and electrodes for increased sensitivity and robust structures. Figure 3 shows illustrative electrical connections of the capacitive sensing electrodes to construct the fully differential capacitive sensing scheme, with insets showing the mechanical connection of the microstructures in the device.

As shown in Figure 3(a), to realize a fully differential sensing scheme by ample use of design rules and material arrangements in MetalMUMPs, the accelerometer proof-mass is separated into three pieces which are mechanically connected by combined layer underneath the main structural nickel layer. The combined layer is comprised of a 0.7 µm polysilicon layer sandwiched by two nitride layers both of which have a thickness of 0.35 µm, as given in Table 1. While being separated from the large piece (1) that serves as an output node of the sensing bridge, the two small diagonal pieces (2) and (3), as shown in Figure 3(a), are connected using a polysilicon layer underneath the nickel layer to form another output node. With this strategy and external swapped wirings, a full sensing bridge can be constructed, as described in the following paragraphs. Figure 3(b) shows the cross-sectional view of the combined layers for the mechanical connection of proof mass pieces (2) and (3). The gap between the two diagonal pieces and the whole large piece are used for mechanical connection and electrical isolation [14].

The inset in Figure 3(a) shows the connection of individual comb finger pairs. Connections of two pairs of the sensing comb drives are illustrated. The two types of stator electrodes that are both anchored to the substrate are electrically isolated from each other. The same type of stator electrodes are electrically connected differently for differential sensing. As shown in the figure, while the two lower stator electrodes in both pairs are directly connected using the nickel metal, the two upper stator electrodes are electrically connected using the doped polysilicon layer underneath the structure material. While the polysilicon layer is isolated from the lower electrodes, it is connected to the upper electrodes in the anchor pads.

**Figure 4.**
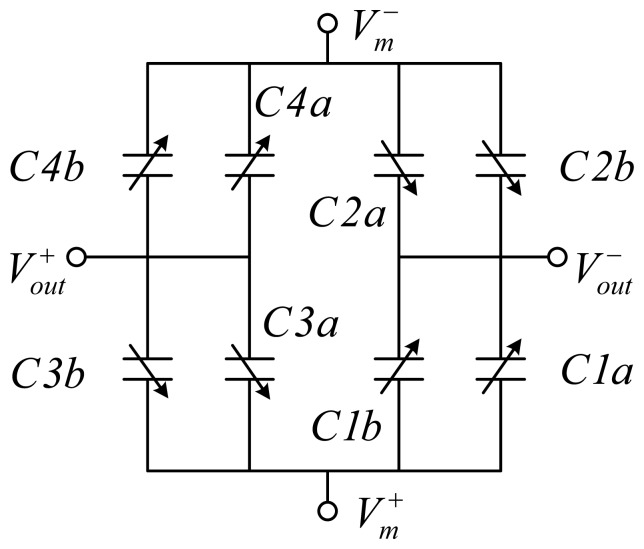
Electrical equivalent circuit for the common-centroid configuration of the sensing capacitors.

A fully differential sensing bridge is formed by wiring the comb fingers in a common-centroid manner as shown in Figure 3(a). *C1a, C3a, C1b* and *C3b* are connected together to positive modulation voltage *V_m_*^+^ while *C2a, C4a, C2b* and *C4b* are connected together to negative modulation voltage *V_m_*^−^ The electrical equivalent circuit of the sensing bridge is shown in Figure 4. A motion of the proof mass in y direction, as shown in Figure 3, has been assumed.

**Figure 5.**
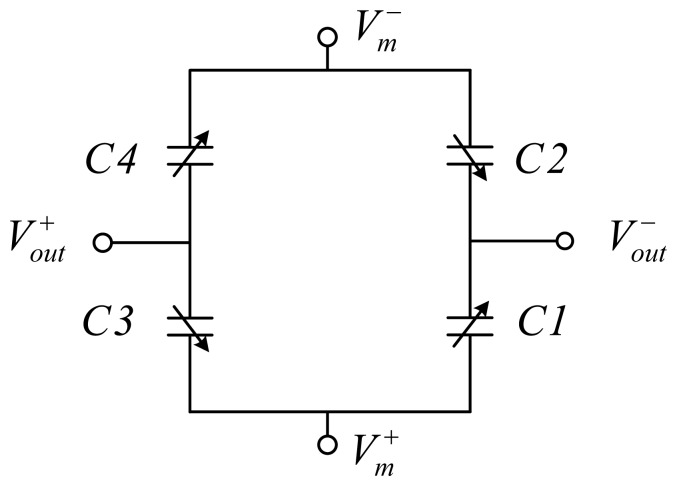
Simplified equivalent circuit of the sensor.

Consequentially, the equivalent circuit in Figure 4 can be further simplified as shown in Figure 5 where *C1* = *C1a* + *C1b, C2* = *C2a* + *C2b, C3* = *C3a* + *C3b* and *C4* = *C4a* + *C4b*.

Referring to Figure 5, the output voltage is given by:

(2) $$V_{s}=\frac{2C_{s}}{2C_{s}+C_{p}}\cdot V_{m}\cdot \frac{y}{y_{0}}$$

where *V_S_* = *V_out_*^+^ − *V_out_*^−^*, C_S_* is the initial sensing capacitance of either of *C1* ~ *C4*, with an calculated value of ~110 fF; *C_P_* is the estimated parasitic capacitance formed by the wiring metal beams and polysilicon. The overall sensitivity, *V_S_* /*a_in_* can be derived as:

(3) $$\frac{V_{s}}{a_{\text{in}}}=\frac{4C_{s}}{2C_{s}+C_{p}}\cdot \frac{1}{\omega^{2}}\cdot \frac{V_{m}}{y_{0}}$$

where *a_in_* is the input acceleration. In this study, due to the suspension of the metal wiring with large gap to the substrate, only the parasitic capacitance resulted from the polysilicon connections are included. A relative permittivity of 4.0 has been used for SiO_2_ underneath the polysilicon connection. The estimated parasitic capacitance for each quarter is ~160 fF; *V_m_* is the modulation voltage; *y* is the displacement of the proof mass under acceleration; *y_0_* is the original finger gap; and *ω* = *2πf* is the angular frequency of mechanical resonance, which can be obtained from the resonant frequency *f*. For a modulation voltage with amplitude of 2.25 V, an overall mechanical sensitivity of 0.71 mV/g can be estimated with the estimated parasitic capacitance.

### 2.3. Device Simulation

**Figure 6.**
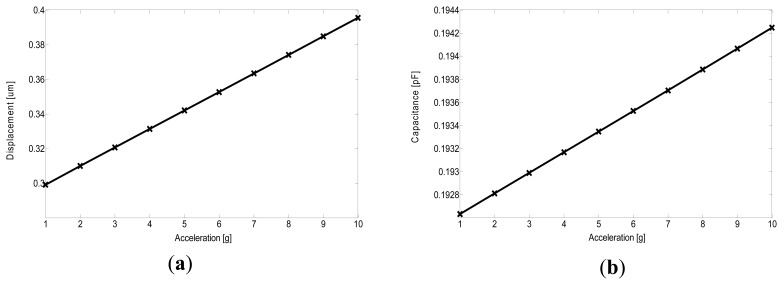
CoventorWare simulation results for displacement (**a**), and capacitance change (**b**), both under acceleration ranging from 1 to 10 g.

CoventorWare, a FEA simulator dedicated to MEMS device design, is used in the structural and electrical design verification for the fully differential accelerometer. The technological parameters presented in the embedded MetalMUMPs process in CoventorWare are used in the whole simulation. As a key mechanical parameter, residual stress of 100 MPa [6] in the nickel structural layer is adopted. Linear responses are observed for both the displacement and capacitance change, as shown in Figure 6. A capacitance sensitivity of ~0.16 fF/g has been obtained. Based on these simulation results, with an external modulation voltage of 2.25 V, a mechanical sensitivity of 2.8 mV/g has been deduced. The results show larger sensitivity than the analytical design value because in FEA simulation, fringe capacitance is included. In addition, the simulator extracts no parasitic effect from the simplified 3-D model. From the modal simulation, the sensor structure demonstrates a resonant frequency of approximately 3.157 kHz, which is within 10% of the calculated value.

## 3. Device Fabrication

**Figure 7.**
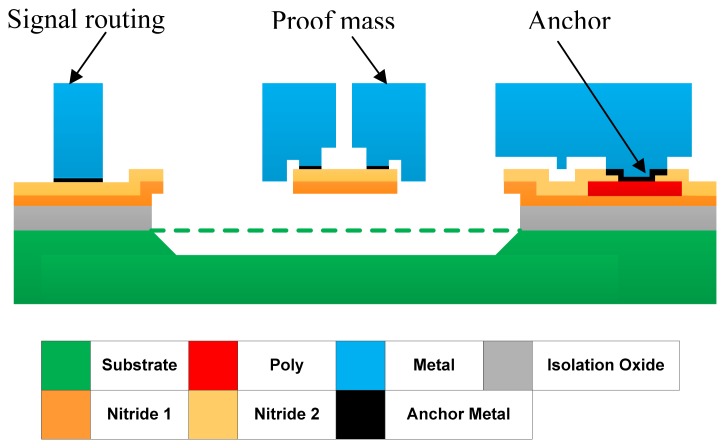
Cross-sectional view of the structures of a released accelerometer with color code for each layer (not to scale) involved. For dimensions of each layer, please refer to Table 1.

The device presented in this paper is fabricated at MEMSCAP using MetalMUMPs technology. In total 10 thin film layers are involved in MetalMUMPs fabrication processes. An illustrative cross-sectional view of the released accelerometer is shown in Figure 7 with color codes showing the thin films involved.

MetalMUMPs technology uses a substrate wafer with high resistivity. A 2 µm layer of silicon oxide (Isolation Oxide) is grown on the entire wafer to provide electrical isolation from the substrate. Layer Nitride 1 in combination with the layer Nitride 2 is used for various purposes in the demonstrated device. Firstly, the combined nitride layers provide a protective encapsulation for the polysilicon that is used for electrical connections of the sensing electrodes and separated proof mass pieces. Secondly, they are patterned to protect other areas in the wet anisotropic Si etching for the trench above which the entire accelerometer structures are suspended. The trench is critical for final device release. Lastly, a patterned nitride area is also used to provide a mechanical connection between the two pieces of proof mass (2) and (3) as shown in Figure (3). This device design makes full use of the insulator layers in MetalMUMPs to achieve a fully differential accelerometer. The 0.7 µm thickness doped polysilicon is mainly used as electrical connection material in the design. It also provides connections in the crosses in the electrical routing. Mechanical structures, including the proof mass and springs, consist of a 20 µm nickel layer and a 0.5 µm gold layer on top of the nickel.

Nickel has been chosen in MetalMUMPs due to its considerably good electrical and elastic properties; and manufacturability using electroplating technology for overall low cost and good process control. The other two layers not shown in Figure 7 are the Oxide 1 and Oxide 2 layers. They are made of phosphosilicate glass (PSG) and act as sacrificial release layers. Layer Oxide 1 in particular is used to define areas where a 25 µm deep trench in the bsilicon substrate will be formed subsequently. It is removed after the releasing Nitride 1 layer process. Oxide 2 is removed by wet chemical etching in the final structural release step to free the entire accelerometer. In the fabrication of the accelerometer in this work, the 25-µm trench underneath the entire device, defined by Oxide 1, is anisotropically etched using KOH after the device is released, and the Oxide 1 is removed.

**Figure 8.**
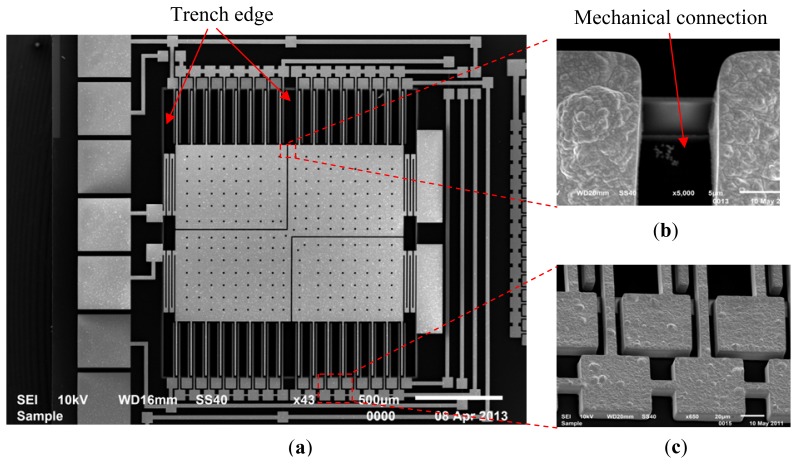
(**a**) The SEM image of a released sensor, (**b**) mechanical connection of proof mass pieces, and (**c**) anchors of the stator sensing fingers.

Figure 8 shows SEM images of a released device with insets showing some detailed microstructures. As shown in the Figure 8(a,b), the proof mass made of nickel is formed by three pieces that are electrically isolated and mechanically connected. Enclosed by the labeled edges, the silicon trench under the proof mass and sensing electrodes can also be seen in the picture. Figure 8(c) shows the anchor structures of the stator electrodes, corresponding to the inset of Figure 3. One group of electrodes is connected directly using structural nickel; the other electrode pads are electrically connected by polysilicon underneath, which is invisible. SEM observations have shown no evidence of in-plane buckling of the comb fingers, although out-of-plane curling is observed, which will be discussed in Section 4.2.

## 4. Device Characterization

### 4.1. Sensor Tests

**Figure 9.**
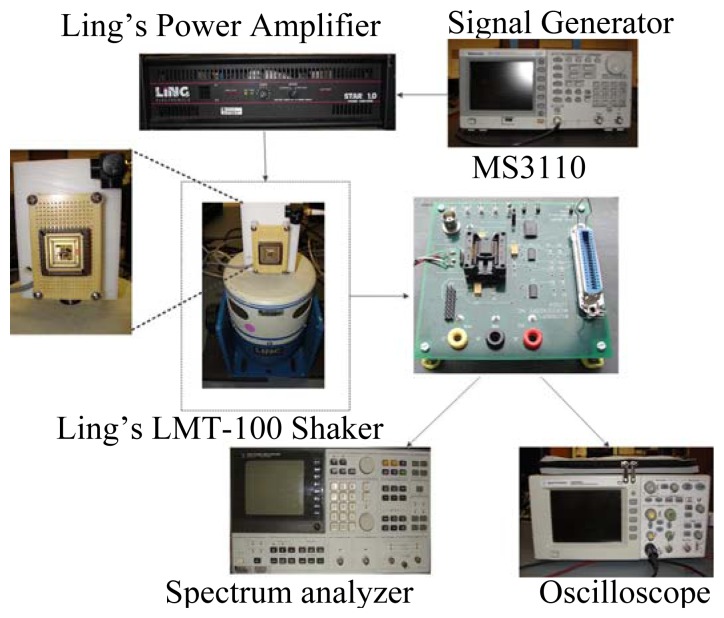
Test setup with inset showing the PCB where the DUT and reference accelerometer are mounted.

In device characterization, a universal capacitive readout circuit IC MS3110 from Irvine Sensors was used. Due to the input configuration of the MS3110 board, only half sensing bridge was connected to the board in each characterization. Yet the swapped bridge was also tested. In circuit configuration and initiation, a modulation voltage of 2.25 V was designed in MS3110. An off-board bandpass filter with a gain of 52 dB was employed for further signal conditioning. Preliminary dynamic tests were conducted using a LMT-100 shaker from Ling Electronics (Corona, CA, USA). In the measurements, a Type 8692B50 PiezoBeam accelerometer from Kistler (Novi, MI, USA) was used as a reference device. Figure 9 shows the test setup and the mounting board on which the MetalMUMPs device under test (DUT) was assembled with the reference accelerometer. The DUT was packaged in a 68 pins J-Bend Leaded Chip Carrier (Evergreen Semiconductor Materials, Inc., San Jose, CA, USA). The board was screwed to the threaded pole of the shaker.

**Figure 10.**
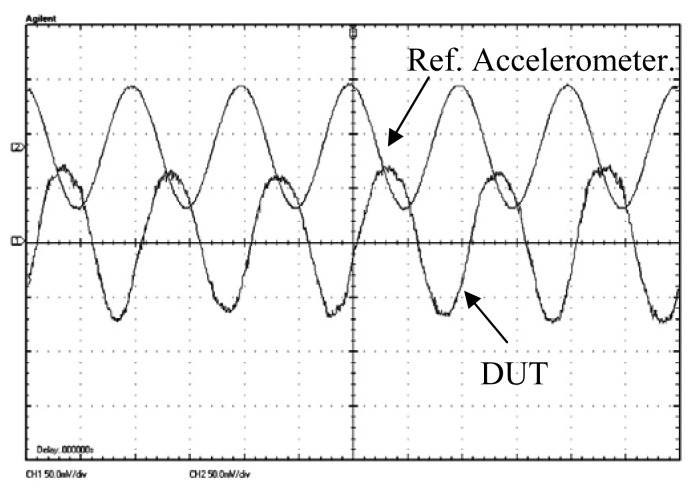
Output waveforms from the DUT and reference accelerometer under a 1 g sinusoidal acceleration. Both channels have the same sensitivity of 50 mV/div.(The phase shift between the two accelerometers is due to the different sensing and parasitic capacitance and readout circuit.)

Figure 10 shows a comparison of the output waveforms made between the fabricated sensor and the reference accelerometer under 1 g acceleration. Prior to the test, the reference accelerometer was calibrated using a PCB Piezoelectronics (Depew, NY, USA) hand-held shaker that can provide standard 1 g acceleration at 159.1 Hz. Due to the design of MS3110 board, each capacitive half-bridge in Figure 5 was tested, respectively. In each test, the excitation was calibrated as a sinusoidal acceleration with amplitude of 1 g and frequency of 110 Hz. The half-bridge sensing scheme demonstrated a sensitivity of ~105 mV/g with a 52 dB external gain, which corresponds to a 0.26 mV/g mechanical sensitivity without external gain. Compared to the designed value, the reduced measured sensitivity is attributed to a few factors including the parasitic capacitance in the test system, increased resonant frequency caused by the residual stress in the mechanical spring, *etc.* The overall sensitivity of the fully differential accelerometer can be demonstrated as ~210 mV/g. The maximum acceleration applied in device characterization is 5 g. Beyond 5 g, the system demonstrates considerable non-linearity that is caused by the mounting of the device and evaluation board on the shaker.

### 4.2. Characterization of Residual Stresses in MetalMUMPs Nickel Layer

Residual stress is a common issue in surface micromachined MEMS devices, as the accelerometer demonstrated in this work. Even though electroplating process is used in nickel deposition, resultant structure deformations caused by residual stresses in the accelerometer structures have been observed in the fabricated device.

When investigating the effects of residual stresses, two types of intrinsic stresses are normally considered, *i.e.*, mean stress and stress gradient [15,16]. The consequence of the residual mean stress in a thin film is compressive or tensile stress along the axial direction of the film after the film release. On the contrary, the consequence of a stress gradient the film thickness direction is the bending of the film structures, such as cantilever beams, upward (positive stress gradient) or downward (negative stress gradient) [11]. Stress characterization of a particular technology is useful to other device and process designs using the same technology. Although some previous works on axial and gradient stress characterization for MetalMUMPs devices have been done by other researchers using special test structures [6,11], it is still worthwhile performing non-destructive stress characterizations directly using the sensing comb drives in the accelerometer. The results presented in this work will provide useful information for design of sensors and actuators such as accelerometers, gyroscopes and electrothermal actuators that are to be fabricated using MetalMUMPs technology.

**Figure 11.**
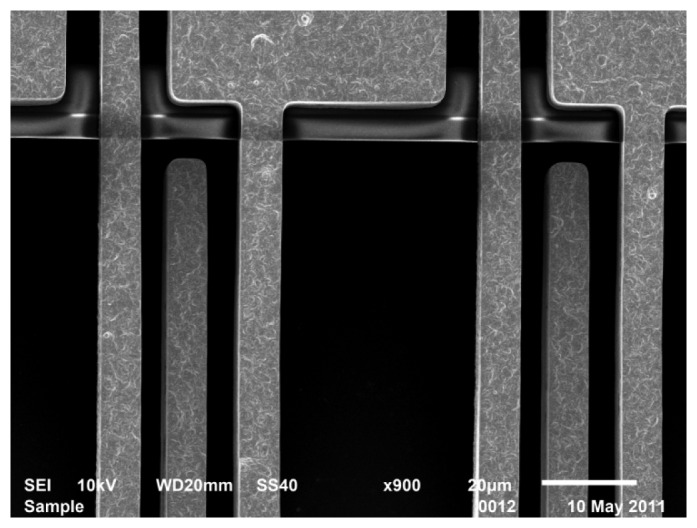
Downward bending can be observed along the sensing comb fingers that are used for stress characterization.

In order to characterize the mean stress and the stress gradient in thin films, different methods have been used to diagnose the states of stress in the film [17]. Among them, measuring the deformations of a cantilever beam made of the thin film to be characterized is considered the simplest and most frequently used method [16,18–20]. In our demonstrated accelerometer, no matter whether they are anchored to substrate or the proof mass, the capacitive sensing fingers can be considered as cantilever beams made of nickel and gold, as shown in Figure 11. It can be observed in SEM scanning that the sensing comb fingers have a downward bending in the fabricated device, which means that a negative stress gradient has been established in device fabrication.

**Figure 12.**
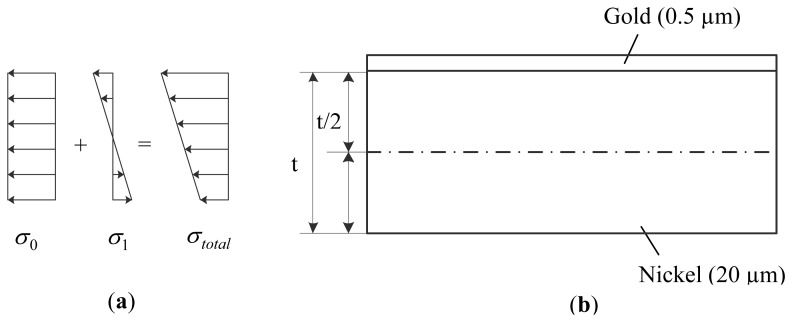
(**a**) Stress distribution in a homogenous cantilever, (**b**) composition of the sensing comb drives in the demonstrated accelerometer.

For the continuous electroplating process in MetalMUMPs, it is reasonable to assume a linear intrinsic stress in the thickness direction of the beam, then the linear stress distribution at a particular location can be expressed as [16,21] :

(4) $$\sigma (z)=\sigma_{0}+\sigma_{1}(\frac{z}{t/2})$$

where *t* is the beam thickness, *z* ∈ (−*t*/*2*, *t*/*2*) is the normal to the surface of the beam with the origin starting at the film's mid plane;*σ_0_* is the residual mean stress for in-plane shrinkage of the beam, and *σ_1_* is the peak value of gradient stress which causes the deflection or curling. The distributions of *σ_0_*, *σ_1_*, and the resultant stress distribution are illustrated in Figure 12(a). The stress distribution leads to a bending moment in the beam given as:

(5) $$M_{0}=\int \sigma (z)\text{wzdz}=\frac{wt^{2}}{6}\sigma_{1}$$

Since the metal structural layer in MetalMUMPs technology is consisted of two layers, *i.e.*, a 20 μm nickel layer with a 0.5 μm gold on the surface, as shown in Figure 12(b), the effective bending moment in the beam can then be expressed as [22]:

(6) $$M_{0}=\frac{{\bar{E}}_{\text{Ni}}I_{\text{Ni}}+{\bar{E}}_{\text{Au}}I_{\text{Au}}}{\rho }$$

where *I_Ni_* and *I_Au_* are the moments of inertia of nickel layer and gold layer respectively about the neutral axis. Because the gold layer is much thinner than the nickel layer, the neutral axis along which zero stress exists can be assumed at the middle of the thickness. In Equation (6),*ρ* is the curvature of the bending beam which can be measured using normally optical methods.

**Figure 13.**
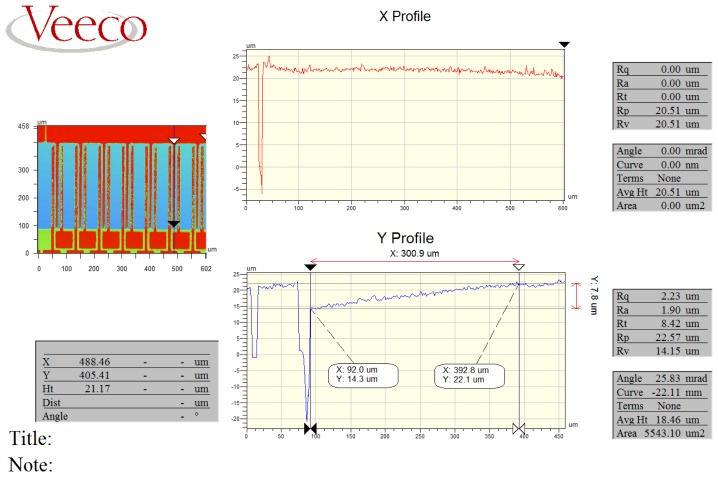
Screen shot of the Veeco post-scanning data processing interface. The data was obtained from a scanning of a group of sensing comb drives at 25 °C.

In this study, a Wyko NT1100 optical profilometer (Tucson, AZ, USA) is used to measure the surface curvature of a deflected sensing comb drive. Stress gradient is then derived based on the measurements. Figure 13 shows a typical measurement data set obtained from NT1100 scanning on some groups of sensing comb drives. Post-scanning data processing allows for extraction of a variety of 3-D geometrical and morphological parameters.

*E̅_Ni_* and *E̅_Au_* are the equivalent Young's moduli of the nickel and gold layer, respectively, which can be expressed as [15]:

(7) $$\bar{E}=\frac{E}{1-\nu }$$

where *E* is the Young's modulus and *ν* is the Poisson ratio. The Young's moduli of nickel and gold are 160 GPa and 79 GPa, while the Poisson ratios of nickel and gold are 0.31 and 0.44, both of which can be obtained from [23].

The two-layered sensing comb drive beams are asymmetrical beams because of the different Young's moduli and thicknesses of the nickel and gold layer. The bending moment caused by the mean residual stress in the nickel layer can be neglected due to the asymmetrical beam [11]. Moreover, the residual stress in gold layer can be neglected due to its much smaller thickness compared to the nickel layer. As the result of the above approximation, the peak value of the gradient stress in the nickel layer can be expressed as:

(8) $$\sigma_{1}=\frac{{\bar{E}}_{\text{Ni}}I_{\text{Ni}}+{\bar{E}}_{\text{Au}}I_{\text{Au}}}{\rho wt^{2}/6}$$

and the in-plane residual mean stress can be determined by:

(9) $$\sigma_{0}={\bar{E}}_{\text{Ni}}\varepsilon ={\bar{E}}_{\text{Ni}}\frac{s-l}{l}$$

where *ε* is the strain of the beam after release; *l* is the original (designed) length of the beam and *s* is the length of the beam after release, which can be deduced by measuring the curvature of the beam obtained in the profile scanning as shown in Figure 13.

With the measured *s* of 300.123 μm and the designed *l* of 300 μm and the effective Young's Modulus of 232 GPa, the stress gradient is calculated as approximately −5.27 MPa/μm using *σ_1_*/(*t*/*2*), where *t* is the beam thickness and the residual mean stress is calculated as 94.67 MPa using Equation (9). These values are within 10% of the values reported in [11]. The differences are due to the variation of each batch.

### 4.3. Capacitance Change Due to the Residual Stresses

Due to the curling of the sensing fingers caused by the residual stress discussed above, the sensing capacitance of each pair is 5.3 fF which differs consequently from the designed value of 6.6 fF that is based on flat comb drives as well.

Without the residual stress, two electrode plates are in parallel with each other. Due to the residual stress, the electrode plates bend downward so that the common area between two electrode plates decrease, the capacitance of the sensing system thus decrease.

**Figure 14.**
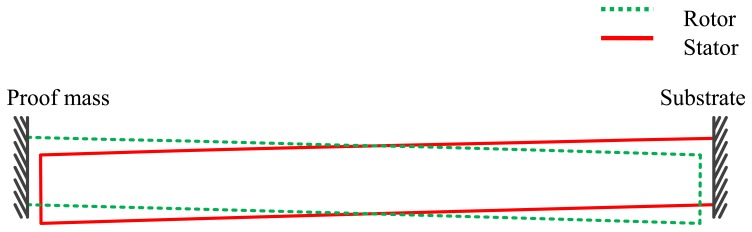
Two symmetric bending electrode plates due to the residual stress.

Assuming the rotor and stator are symmetric, the two bending electrode plates due to the residual stress are shown in Figure 14. Because the bending distance is much smaller in comparison with the length of the sensing finger, the surface profile of electrode plates is approximated as straight line instead of curve. In this case, the common area of two electrode plates after release is about 1/6 less, thus the total capacitance of the accelerometer decreases 1/6 of the designing value.

### 4.4. Temperature Dependence of the Sensing Capacitance

Temperature dependences of the sensing capacitance and resultant parameters have been also characterized. A Kapton micro heater from Omega (Stamford, CT, USA) is attached to the backside of the device package to heat the sensor structure, which is placed underneath the objective lens of Veeco surface profilometer. Profiles of the comb drive surface at three temperature points, *i.e.*, 25, 50 and 73 °C, are scanned respectively.

**Figure 15.**
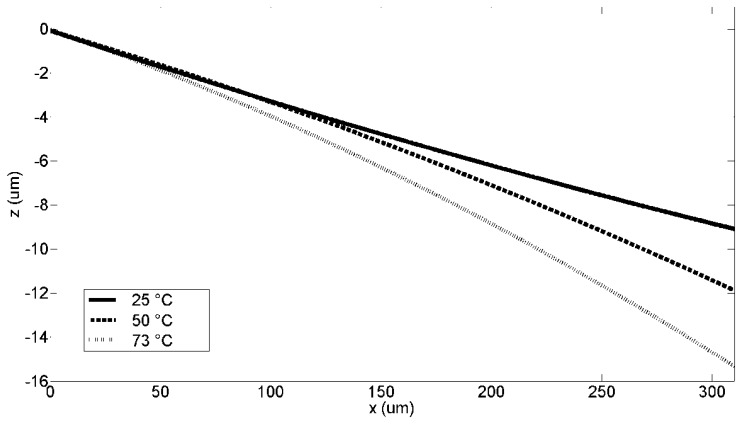
Quadratic fitted surface profiles of a sensing finger extracted from the original Veeco data at 25, 50 and 73 °C, respectively.

Figure 15 depicts fitted surface profile of a sensing finger extracted from the original Veeco data at temperatures of 25, 50 and 73 °C, respectively. Note the anchored ends are normalized to the same height. The downward bending with elevated temperature, as revealed by the profiles, results in a −0.021 fF/°C of the temperature dependence of the capacitance (TDC). It corresponds to a 10% and 19% reduction at 50 °C, and 73 °C, compared to the capacitance of each sensing pair at 25 °C, respectively. Based on the above TDC, a temperature coefficient of sensitivity (TCS) of −0.014 mV/°C can be derived. No apparent in-plane buckling is observed.

## 5. Conclusions

A capacitive accelerometer enabled by MetalMUMPs foundry technology has been designed, fabricated and characterized in this project. The device features a fully differential sensing scheme with a unique common-centriod capacitance configuration. In simulation results, linear responses for both displacement and capacitance are observed. With a ~52 dB external amplification gain, the accelerometer achieves a sensitivity of ~210 mV/g. Without amplification, the device demonstrates a mechanical sensitivity of 0.52 mV/g. Residual mean stress and stress gradient are characterized as 94.67 MPa and −5.27 MPa/μm. Respectively, by measuring the surface profile using a Wyko NT1100 optical surface system. The temperature dependence of the sensing capacitance is characterized as −0.021 fF/°C, which results in a temperature coefficient of sensitivity of −0.014 mV/°C. No apparent in-plane buckling is observed. The data reported in this paper provides other MetalMUMPs user an important reference for designing and optimizing suspended MEMS device.

## Figures and Tables

**Table 1.** Structural parameters of the device.

**Layers**	**Thickness (μm)**
Isolation Oxide	2
Oxide 1	0.5
Nitride 1	0.35
Poly	0.7
Oxide 2	1.1
Nitride 2	0.35
Anchor Metal	0.035
Nickel structural layer	20
Gold coating layer	0.5

**Table 2.** Geometric and material properties.

**Symbols**	**Description**	**Values**
*E*	Nickel's Young's Modulus (GPa)	160 [11,12]
*Lm* × *Wm*	Proof mass length and width (µm × µm)	1,000 × 1,300
*Ls*	Sensing finger length (µm)	300
*Ws*	Sensing finger width (µm)	10
*t*	Structure thickness (µm)	20
*L* × *W*	Springs length and width (µm × µm)	1,200 × 8
*g*	All sensing finger gaps (µm)	8.0
*N*	Number of sensing fingers	16 × 4
